# Positron Emission Tomography with [^18^F]ROStrace Reveals Progressive Elevations in Oxidative Stress in a Mouse Model of Alpha-Synucleinopathy

**DOI:** 10.3390/ijms25094943

**Published:** 2024-05-01

**Authors:** Evan Gallagher, Catherine Hou, Yi Zhu, Chia-Ju Hsieh, Hsiaoju Lee, Shihong Li, Kuiying Xu, Patrick Henderson, Rea Chroneos, Malkah Sheldon, Shaipreeah Riley, Kelvin C. Luk, Robert H. Mach, Meagan J. McManus

**Affiliations:** 1 Department of Anesthesia and Critical Care Medicine, Children’s Hospital of Philadelphia, Philadelphia, PA 19104, USA; evangall@pennmedicine.upenn.edu (E.G.);; 2 Department of Radiology, University of Pennsylvania, Philadelphia, PA 19104, USA; houc@pennmedicine.upenn.edu (C.H.); rmach@pennmedicine.upenn.edu (R.H.M.); 3 Department of Pathology and Laboratory Medicine, Institute on Aging and Center for Neurodegenerative Disease Research, Perelman School of Medicine, University of Pennsylvania, Philadelphia, PA 19104, USA

**Keywords:** alpha-synuclein, oxidative stress, reactive oxygen species, PET imaging, Parkinson’s disease, synucleinopathy, A53T, M83

## Abstract

The synucleinopathies are a diverse group of neurodegenerative disorders characterized by the accumulation of aggregated alpha-synuclein (aSyn) in vulnerable populations of brain cells. Oxidative stress is both a cause and a consequence of aSyn aggregation in the synucleinopathies; however, noninvasive methods for detecting oxidative stress in living animals have proven elusive. In this study, we used the reactive oxygen species (ROS)-sensitive positron emission tomography (PET) radiotracer [^18^F]ROStrace to detect increases in oxidative stress in the widely-used A53T mouse model of synucleinopathy. A53T-specific elevations in [^18^F]ROStrace signal emerged at a relatively early age (6–8 months) and became more widespread within the brain over time, a pattern which paralleled the progressive development of aSyn pathology and oxidative damage in A53T brain tissue. Systemic administration of lipopolysaccharide (LPS) also caused rapid and long-lasting elevations in [^18^F]ROStrace signal in A53T mice, suggesting that chronic, aSyn-associated oxidative stress may render these animals more vulnerable to further inflammatory insult. Collectively, these results provide novel evidence that oxidative stress is an early and chronic process during the development of synucleinopathy and suggest that PET imaging with [^18^F]ROStrace holds promise as a means of detecting aSyn-associated oxidative stress noninvasively.

## 1. Introduction

Alpha-synuclein (aSyn) is a 140 amino acid protein that is highly enriched in the presynaptic terminals of mature neurons [1,2,3]. Despite decades of focused research, the diverse physiological functions of aSyn within the cell remain incompletely understood, with various studies demonstrating roles for aSyn in the regulation of exo- and endocytosis [4,5,6,7], vesicle trafficking and recycling [8,9,10], dopamine metabolism and homeostasis [11], DNA/RNA expression and repair [12], mitochondrial calcium homeostasis [13], and the assembly of soluble N-ethylmaleimide-sensitive factor activating protein receptor (SNARE) complexes that mediate membrane fusion events [14]. Similarly, the structural properties of native aSyn are not fully characterized, in large part because aSyn’s structure is thought to be both dynamic and context-dependent [15,16,17,18]. Although the structure and function of aSyn under healthy conditions remain topics of intense scientific interest, it is well-established that misfolding and aggregation of aSyn plays a central role in the pathogenesis of several prominent neurodegenerative disorders, including Parkinson’s disease (PD), dementia with Lewy bodies (DLB), and multiple system atrophy (MSA), among others. These disorders—which are collectively labeled synucleinopathies—are characterized by the aberrant accumulation of insoluble, aSyn-rich inclusions in vulnerable populations of neurons or glial cells. While aSyn aggregation is associated with cytotoxicity [19], it has long been recognized that aSyn-associated neurodegeneration may precede clinical symptom presentation by years or even decades [20]. This extensive preclinical (i.e., prodromal) phase may represent a critical window for disease-altering interventions; therefore, noninvasive biomarkers that can identify and track disease progression in the absence of clinical symptoms are needed.

While the biological mechanisms underlying aSyn aggregation and aSyn-induced toxicity remain incompletely understood, a substantial body of evidence suggests that oxidative stress—i.e., an imbalance in the production and clearance of reactive oxygen species (ROS) such as superoxide, hydrogen peroxide, and the hydroxyl radical—is both a cause and a consequence of aSyn aggregation in synucleinopathies. For example, it is well-established that insoluble Lewy bodies (LBs) from PD brain tissue contain oxidatively modified aSyn, whereas soluble aSyn generally does not bear similar markers of ROS-induced damage [21]. Likewise, animal studies in primates [22] and rodents [23] have demonstrated that neurotoxin-induced oxidative stress promotes the formation of insoluble inclusions that contain oxidatively modified aSyn, and more recent studies have shown that oxidizing conditions enhance both the formation and intracellular propagation of aSyn pathology [24,25]. Additional in vitro experiments support a direct causal link between increased levels of ROS and enhanced aSyn aggregation. For instance, exposure of aSyn-expressing cells to oxidants such as peroxynitrite has been shown to induce the formation of insoluble aSyn inclusions [26], supporting the idea that ROS and reactive nitrogen species (RNS) interact with aSyn in a manner that promotes aggregation. Critically, aSyn aggregates themselves can also induce ROS production through a variety of mechanisms, including disrupting mitochondrial complex I activity [27,28] and inducing microglial activation [29,30]. Indeed, oxidatively modified aSyn has been shown to be more toxic to dopaminergic neurons than wild-type aSyn [31] and elicit a more severe immune response than wild-type aSyn [32]. The cyclical relationship between aSyn aggregation and increased ROS production suggests that ROS could be leveraged as predictive biomarkers of disease progression; however, the utility of ROS as biomarkers is currently limited by the lack of noninvasive methodologies for detecting them in vivo.

The positron emission tomography (PET) radiotracer [^18^F]ROStrace detects changes in ROS production in vivo [33,34] and therefore holds promise as a means by which to identify and track aSyn-associated oxidative stress noninvasively. When [^18^F]ROStrace is injected intravenously, its lipophilicity and neutral charge allow it to cross the blood–brain barrier and enter the brain parenchyma. Subsequently, [^18^F]ROStrace is oxidized by ROS to [^18^F]ox-ROStrace, which is unable to exit the brain because of its positive charge. Previous imaging studies in rodent models of neuroinflammation and neurodegeneration have shown that elevations in ROS production result in increased trapping of [^18^F]ox-ROStrace in brain, and therefore in higher brain PET signal [33,34,35]. However, this methodological platform has not yet been assessed in the context of aSyn aggregation. To address this knowledge gap, we utilized the widely studied M83 mouse line, which overexpresses the A53T mutant form of the human aSyn protein under the control of the mouse prion promoter [36]. A53T aSyn results from a G209A base-pair change in the *SNCA* gene, and expression of A53T aSyn in humans is associated with an early and rapidly progressing form of familial PD [37]. Likewise, A53T mice are known to show age-dependent accumulation of aSyn pathology throughout the spinal cord, brainstem, and brain, and as such we hypothesized that A53T animals would show higher average [^18^F]ROStrace signal in brain compared with age-matched B6C3 controls. Furthermore, we postulated that A53T-specific elevations in [^18^F]ROStrace signal would become detectable prior to the onset of debilitating late-stage motor symptoms in A53T mice. To test these hypotheses, we performed [^18^F]ROStrace PET imaging on A53T and B6C3 mice at early (6–8 months old; mo) and middle (12 mo) stages of aSyn aggregation. These studies demonstrated that average [^18^F]ROStrace signal is elevated in A53T mice, coincident with neurobehavioral changes at 6–8 mo and the development of phosphorylated aSyn pathology in A53T brain. Furthermore, A53T-specific elevations in [^18^F]ROStrace signal became progressively larger and more widespread over time, and consistently appeared well before the emergence of the late-stage ataxic phenotype that is characteristic of the A53T model. To evaluate the predictive power of [^18^F]ROStrace PET for aSyn-dependent nigrostriatal degeneration [38], additional cohorts of A53T and B6C3 mice were injected with lipopolysaccharide (LPS) at 7 mo and imaged either 24 h, 1 m, or 5 m after the injection alongside saline-injected controls. In these studies, LPS-injected A53T mice showed both acute and chronic elevations in whole-brain [^18^F]ROStrace signal compared with LPS-injected B6C3 mice, coincident with increased sickness and mortality rates in LPS-injected A53T animals. Together, these results support the idea that oxidative stress is elevated early in synucleinopathy and highlight the utility of [^18^F]ROStrace PET as a means of detecting aSyn-associated oxidative stress noninvasively.

## 2. Results

### 2.1. Corpus Callosum Is the Most Appropriate Pseudo-Reference Region for [^18^F]ROStrace Quantification in A53T Mice

To measure levels of oxidative stress in presymptomatic A53T mice, we conducted 60 min dynamic [^18^F]ROStrace PET imaging experiments on cohorts of 6–8 mo and 12 mo A53T and B6C3 animals of both sexes. While the gold-standard approach to PET image quantification involves the collection of arterial blood samples at pre-established timepoints throughout each scan, mice do not have sufficient blood volume to enable this approach. Previous small animal PET studies have surmounted this challenge by normalizing the PET signal to that of a reference region—i.e., a volume of interest (VOI) within the brain that is known to show no specific uptake of the radiotracer [39,40,41,42]. However, ROS are continuously produced in brain cells as byproducts of cellular respiration [43] and are also important contributors to redox signaling even under normal physiological conditions [44]. As such, no major brain region is likely to show zero specific [^18^F]ROStrace signal. In light of this, we opted to normalize the [^18^F]ROStrace signal to that of a pseudo-reference region, which is a region in the brain that is known to be unaffected by the disease or process being studied (in this case, aSyn aggregation and any associated increases in ROS production) [45,46]. To identify a region that is unaffected by aSyn aggregation and aSyn-associated oxidative stress in presymptomatic A53T mice, we immunostained brain sections from 8–16 mo A53T animals with multiple established markers of protein aggregation and oxidative stress (Figure S1). These studies revealed that the corpus callosum (CC) is the brain region least affected by aSyn aggregation and aSyn-associated oxidative stress in the A53T model (further details available in the Methods Section). As such, a CC VOI was added to the M. Mirrione mouse brain atlas [47], and each [^18^F]ROStrace PET image was normalized to the average signal in the corresponding CC. This resulted in a final PET outcome measure of standardized uptake value relative to CC (SUVRcc).

### 2.2. A53T Mice Show Progressive Increases in Brain [^18^F]ROStrace Signal

**Figure 1:**
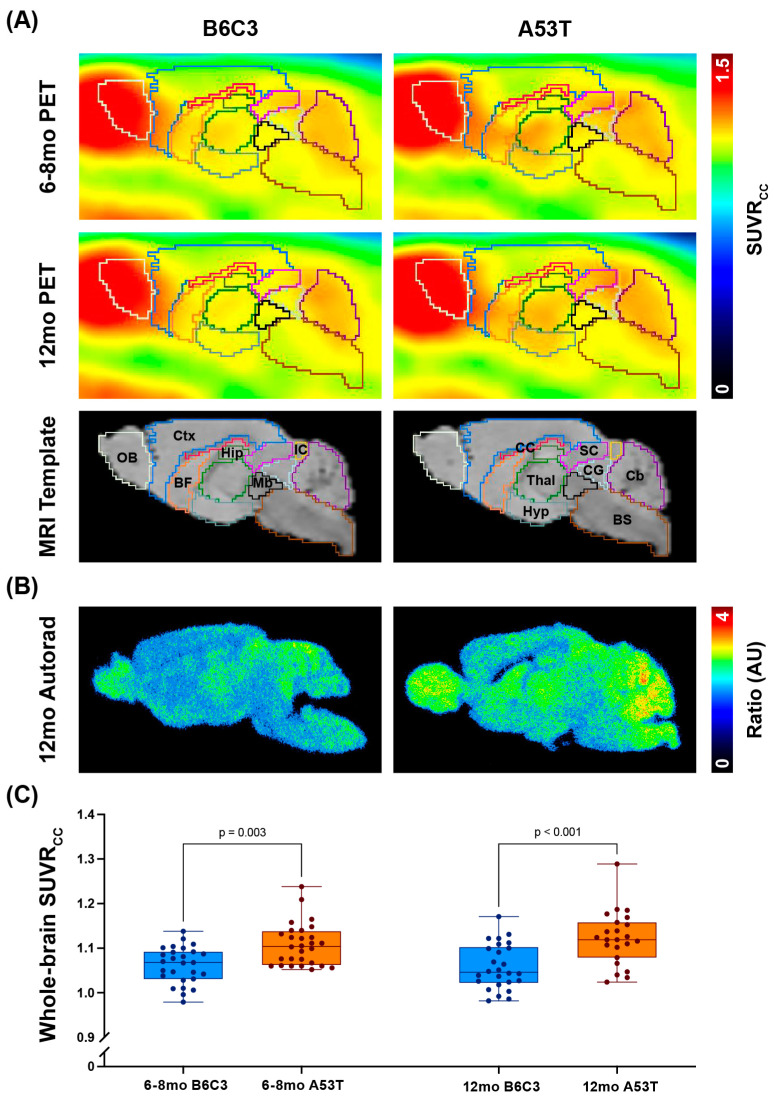
Whole-brain average [^18^F]ROStrace signal is elevated in A53T mice. (**A**) Average brain [^18^F]ROStrace PET images from B6C3 and A53T mice at 6–8 and 12 months old (mo). In all PET images, colors represent unitless SUVRcc values, which are calculated by dividing each animal’s raw PET image by the average signal in the corpus callosum (CC). Brain subregions are overlayed onto each PET image, with corresponding labels shown in the MRI Template images; OB = olfactory bulb, Ctx = cortex, BF = basal forebrain and septum, CC = corpus callosum, Thal = thalamus, Hyp = hypothalamus, Hip = hippocampus, Mb = midbrain, SC = superior colliculi, IC = inferior colliculi, CG = central grey, BS = brainstem, Cb = cerebellum. (**B**) Representative ex vivo autoradiography images from 12 mo male B6C3 and A53T animals ~60 min after [^18^F]ROStrace injection. As in the PET images, these autoradiographs are normalized to the average signal in the corpus callosum. (**C**) Whole-brain SUVRcc values in B6C3 and A53T mice at each timepoint. Mean values were as follows: 6–8 mo B6C3 = 1.062; 6–8 mo A53T = 1.108; 12 mo B6C3 = 1.058; 12 mo A53T = 1.122. *p*-values were calculated via one-way ANOVA with post hoc Šídák’s multiple comparisons tests.

Since previous [^18^F]ROStrace studies in mice have demonstrated that tracer uptake in brain peaks within 2–3 min and then plateaus around 40 min [34,35], we compared the average SUVRcc in brain from 40–60 min across A53T and B6C3 animals at both timepoints. As expected, A53T mice showed significantly higher whole-brain SUVRcc than B6C3 mice regardless of age (Figure 1; *p* ≤ 0.003). These results were corroborated by ex vivo autoradiography studies, which showed a similar difference between A53T and B6C3 animals when the raw images were normalized to the average intensity in corpus callosum (Figure 1B). While both male and female animals were scanned at each timepoint (sample sizes shown in Table S1), no significant sex effect was observed in either genotype (A53T male vs. A53T female *p* = 0.699; B6C3 male vs. B6C3 female *p* = 0.408; *p*-values calculated via one-way ANOVA with Šidák’s multiple comparisons test).

**Figure 2:**
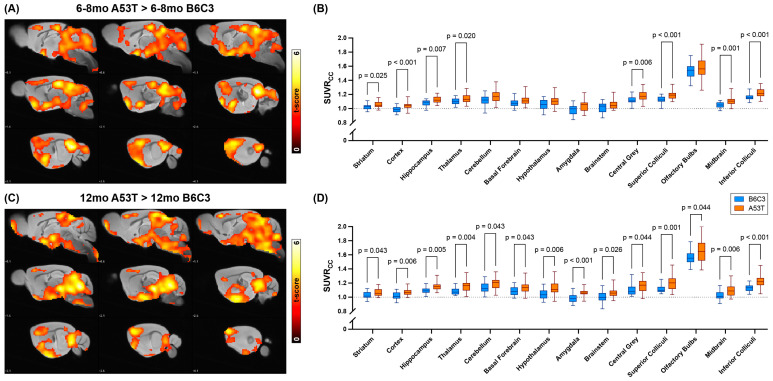
A53T-specific elevations in [^18^F]ROStrace signal become more widespread with age. (**A**) Statistical parametric mapping (SPM) images showing voxels that have significantly higher average SUVRcc in 6–8 mo A53T mice vs. 6–8 mo B6C3 mice. Hotter colors correspond to higher t-scores (i.e., lower *p*-values) and significant clusters are overlayed onto a standard mouse brain MRI. The right hemisphere is shown; *p* < 0.005 uncorrected; extent threshold = 50. (**B**) Subregion-specific SUVRcc values from 6–8 mo B6C3 (blue) and A53T (orange) mice. *p*-values were calculated via multiple unpaired t-tests with the Holm–Šidák method of multiple comparison correction, and only values less than 0.05 are shown. A dashed line at SUVRcc = 1 is also added for visual clarity. (**C**,**D**) Corresponding SPM images (**C**) and subregional SUVRcc results (**D**) from 12 mo B6C3 and A53T mice. Notably, every subregion showed significant A53T > B6C3 differences in [^18^F]ROStrace signal at the 12 mo timepoint, including those that did not show such elevations at the 6–8 mo timepoint.

Having established that A53T mice show higher whole-brain [^18^F]ROStrace signal than B6C3 mice at both 6–8 mo and 12 mo, we next investigated how the spatial distribution of [^18^F]ROStrace signal differences changed over time (Figure 2). Voxel-level statistical comparisons between the A53T and B6C3 SUVRcc image sets were first performed using statistical parametric mapping (SPM; Figure 2A,C). These SPM results revealed widespread A53T-specific elevations in SUVRcc at both timepoints, with cortex, hippocampus, thalamus, striatum, and midbrain showing clusters of significant A53T > B6C3 differences at both 6–8 mo and 12 mo. Notably, several additional regions only showed significant differences at 12 mo, including brainstem, deep cerebellar grey matter, and olfactory bulbs, among others. To further clarify which regions showed significant elevations in [^18^F]ROStrace signal at each timepoint, we next overlayed the M. Mirrione mouse brain atlas [47] onto each PET image and calculated the average SUVRcc in 14 VOIs throughout the brain. The resulting region-specific SUVRcc values were then compared across genotypes at each timepoint (Figure 2B,D). As expected based on the SPM results, A53T mice showed higher average SUVRcc than B6C3 mice in a subset of brain regions at 6–8 mo, and in every brain region at 12 mo. These VOI-level results align with the voxel-level results shown in the SPM images and indicate that A53T-specific elevations in [^18^F]ROStrace signal become more widespread within the brain as the animals age.

### 2.3. A53T Mice Exhibit Progressively Severe aSyn Pathology That Colocalizes with Indicators of Oxidative Stress

**Figure 3:**
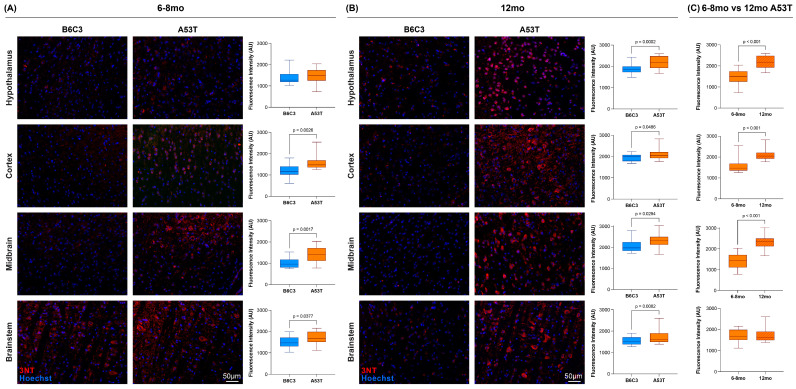
A53T mice show progressive increases in tyrosine nitration relative to age-matched B6C3 mice. (**A**) Representative immunofluorescent images of 3-nitrotyrosine (3NT) staining in various regions of 6–8 mo B6C3 (**left**) and A53T (**middle**) mouse brain. In all images, 3NT staining (red) shows tyrosine residues that have been nitrated by reactive oxygen and nitrogen species, while Hoechst staining (blue) shows cell nuclei. Box-and-whisker plots (**right**) quantify the 3NT staining shown in each region, with *p*-values calculated via two-tailed unpaired *t*-tests. (**B**) Equivalent images and graphs from 12 mo animals. (**C**) Box-and-whisker plots comparing the 3NT fluorescence of 6–8 mo and 12 mo A53T animals. Sample sizes for staining quantification are as follows: 6–8 mo B6C3 = 4, 6–8 mo A53T = 4, 12 mo B6C3 = 6, 12 mo A53T = 5.

To determine whether increased [^18^F]ROStrace signal is associated with increased histological evidence of oxidative stress, we next stained 6–8 and 12 mo A53T and B6C3 brain sections with antibodies against 3-nitrotyrosine (3NT). Nitration of tyrosine residues is a well-established biomarker of oxidative and nitrative stress [48,49,50,51], and increased tyrosine nitration has consistently been observed in both human synucleinopathy [21,52] and in various animal models of genetic- and toxin-induced neurodegeneration [23,35,53,54,55,56]. As expected based on the PET results, A53T animals showed 3NT staining throughout the brain at both timepoints (Figure 3). At the 6–8 mo timepoint (Figure 3A), average 3NT fluorescence was higher in A53T animals than in B6C3 animals in cortex, midbrain, and brainstem, but not in hypothalamus. This result aligns with the VOI-level [^18^F]ROStrace PET results at this timepoint (Figure 2B). The 3NT staining results at 12 mo also align with the [^18^F]ROStrace PET results, as every examined brain region showed significantly higher 3NT fluorescence in 12 mo A53T animals compared with 12 mo B6C3 animals (Figure 3B). When the 3NT staining in 12 mo A53T animals was compared with that in 6–8 mo A53T animals, most brain regions showed significantly higher signals in 12 mo animals (Figure 3C), consistent with the idea that aSyn-associated oxidative stress becomes progressively severe over time. Taken together, these 3NT staining results support the in vivo PET results and suggest that [^18^F]ROStrace imaging provides a viable means by which to assess changes in oxidative stress noninvasively.

**Figure 4:**
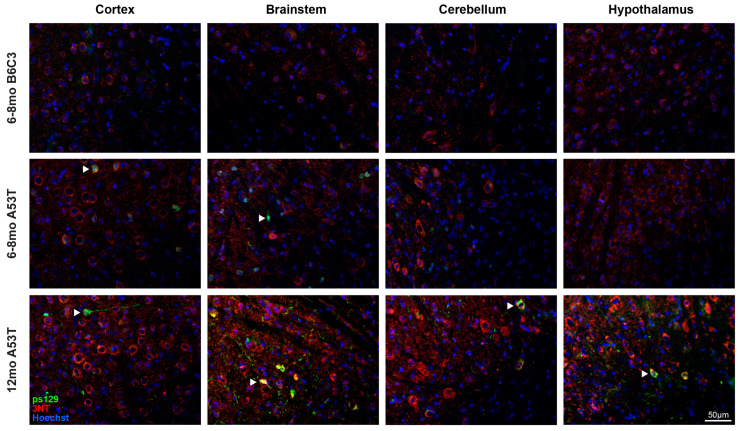
Phosphorylated aSyn pathology becomes more widespread over time and colocalizes with oxidative damage in A53T mouse brain. All panels show representative immunofluorescent images of 6–8 mo B6C3 (**top**), 6–8 mo A53T (**middle**), or 12 mo A53T (**bottom**) brain sections stained with phospho-serine 129 (ps129) and 3-nitrotyrosine (3NT). In all images, ps129 staining (green) shows phosphorylated aSyn pathology, 3NT staining (red) shows evidence of oxidative stress, and Hoechst staining (blue) shows cell nuclei. Diffuse aSyn pathology was detectable in the cortex and brainstem of 6–8 mo A53Ts, whereas 12 mo A53Ts showed bright and distinct pathology in all examined brain regions. B6C3 animals did not show ps129 staining at any timepoint. In samples with positive ps129 staining, the majority of the ps129 signal was found in 3NT-positive cells (examples indicated by white arrowheads; higher magnification images shown in Figure S2).

Having established that A53T animals show significant elevations in both [^18^F]ROStrace PET signal and 3NT fluorescence, we next sought to characterize the spatial associations between these oxidation biomarkers and aSyn pathology. To this end, we first co-stained 6–8 and 12 mo A53T and B6C3 brain sections with 3NT and phospho-serine 129 (ps129), a selective marker of pathological aSyn aggregates in human LBs [57,58,59,60] (Figure 4). As expected, these studies revealed phosphorylated aSyn pathology in A53T brain sections at both timepoints, as well as an absence of pathology in all B6C3 brain sections. Regardless of age, ps129-labeled aSyn aggregates were predominantly found in 3NT-positive cells (examples indicated by white arrowheads in Figure 4; higher magnification images shown in Figure S2), supporting the idea that aSyn pathology is spatially associated with oxidative stress at the cellular level. Likewise, in brain samples co-stained with ps129 and the ROS-sensitive fluorescent probe dihydroethidium (DHE), ps129-positive inclusions were exclusively found in DHE-positive neurons (Figure S1H), providing further evidence that phosphorylated aSyn aggregates colocalize with fluorescent indicators of oxidative stress in A53T brain tissue. While the spatial associations between ps129 and 3NT/DHE were highly consistent across A53T brain samples regardless of age, we observed that the nature and distribution of the ps129 signal differed dramatically between 6–8 mo and 12 mo A53T animals. In younger A53T mice, ps129 fluorescence was relatively dim, predominantly perikaryal, and localized primarily to the superficial layers of cortex, superior and inferior colliculi, grey matter of cerebellum, olfactory bulb, and caudal brainstem/cervical spinal cord. This pattern of staining was highly consistent across all 6–8 mo A53T brains in this study (*n* = 11), and no such staining was observed in age-matched B6C3 brains. By contrast, most 12 mo A53T animals showed ps129-positive inclusions in all major brain regions except CC, including the regions spared by ps129 pathology at 6–8 mo (e.g., hypothalamus and deep cerebellar grey matter; Figure 4). In general, the ps129 signal in 12 mo A53Ts was most abundant in midbrain, cortex, hypothalamus, cerebellum, and brainstem, a pattern which aligned with the distribution of [^18^F]ROStrace signal differences at this timepoint (Figure 2C).

To further characterize the cellular localization of aSyn aggregates in A53T brains at each timepoint, we next co-stained 6–8 and 12 mo A53T brain sections with ps129 and antibodies against NeuN (neurons), ionized calcium-binding adaptor molecule 1 (Iba1; microglia), glial fibrillary acidic protein (GFAP; astrocytes), or SRY-box transcription factor 10 (Sox10; oligodendrocytes—Figure S3). These studies revealed that the vast majority (~80%) of the ps129 signal was found in neurons, with a much smaller fraction (~20%) found in oligodendrocytes and an even smaller fraction (<1%) found in microglia. Negligible ps129 was present in astrocytes at either timepoint. In most A53T animals, the ps129 signal in neurons was localized predominantly to the cell body, although some 6–8 mo A53Ts did show neurite-like pathology in and around the olfactory bulb, and the most severely affected 12 mo A53Ts showed axonal and/or dendritic pathology throughout the brain (e.g., the 12 mo A53T shown in Figure 4). Notably, Iba1 staining revealed minimal microglial activation in the vast majority of A53T mice, regardless of age (Figure S4). Indeed, activated microglia were only observed in acutely symptomatic animals, suggesting that A53T mice generally do not show widespread microgliosis until the onset of fatal motor symptoms in the last 2–3 weeks of life.

### 2.4. A53T-Specific Behavioral Abnormalities Emerge Early in Life and Persist through Middle Age

**Figure 5:**
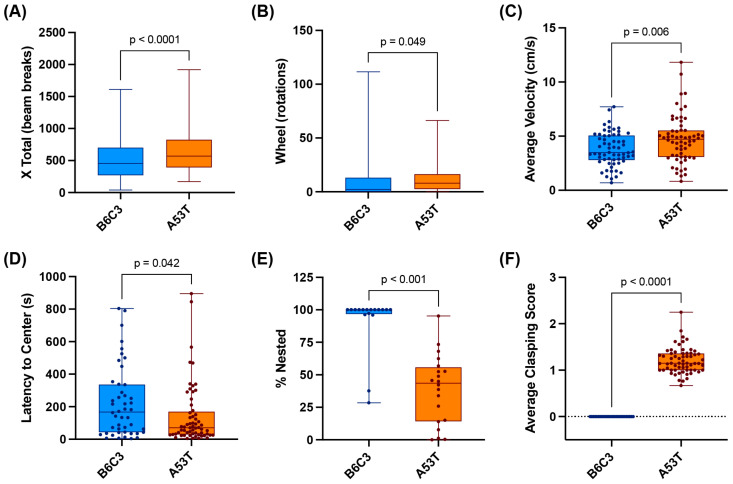
A53T mice display an abnormal and persistent behavioral phenotype characterized by hyperactivity, limb clasping, and disruptions to nesting behavior. (**A**,**B**) A53T mice display increased locomotion (**A**) and running wheel usage (**B**) over the course of 3–4 nights in the Comprehensive Lab Animal Monitoring System (CLAMS); B6C3 *n* = 11, A53T *n* = 11, *p*-values calculated via unpaired *t*-tests. (**C**,**D**) A53T mice show higher average velocity (**C**) and lower average latency to center (**D**) in the open field task; B6C3 *n* = 34, A53T *n* = 38. (**E**) A53T mice show reduced nesting behavior during the nesting task; B6C3 *n* = 17, A53T *n* = 20. (**F**) A53T mice show significant elevations in clasping behavior relative to age-matched B6C3 mice. Each dot on this graph shows the average clasping score for an individual animal; B6C3 *n* = 27, A53T *n* = 63. In all graphs, animal ages range from 6–12 mo.

In human synucleinopathy, the presentation of clinical motor symptoms is often preceded by an extensive ‘prodromal’ phase characterized by a variety of nonmotor symptoms and/or subtle disruptions to normal motor function [20]. Given our observation that A53T animals showed significant elevations in whole-brain average [^18^F]ROStrace signal well before the onset of fatal end-stage motor symptoms (~12–16 mo in our A53T colony), we next sought to determine whether A53T mice also show behavioral evidence of a subacute, ‘prodromal-like’ disease state. Towards this end, we conducted a variety of behavioral assays on A53T and B6C3 mice at both 6–8 and 12 mo. These assays revealed a subtle but highly consistent A53T-specific behavioral phenotype characterized by hyperactivity, diminished nesting behavior, and increased limb clasping (Figure 5). Notably, this phenotype did not become progressively more severe over time; rather, A53T-specific behavioral disruptions developed at an early age (~6 mo in most animals) and then remained relatively stable until the sudden and dramatic loss of function in the final 2–3 weeks of the A53T lifespan. Because we did not observe major differences in behavior between the 6–8 and 12 mo timepoints, and because previous studies in A53T mice have noted a similar plateau in motor symptom development [61], we ultimately chose to combine the behavioral data from both timepoints.

To evaluate gross locomotor activity in A53T and B6C3 mice, we first housed cohorts of male and female animals in the Comprehensive Lab Animal Monitoring System (CLAMS). These studies consistently revealed higher motor activity in A53T animals compared with B6C3 animals, as determined by both total distance traveled (‘X total’; Figure 5A) and running wheel usage (‘wheel’; Figure 5B). These results were corroborated by the open field task, which also showed a higher average velocity (Figure 5C) and longer distance traveled in A53T mice compared with B6C3 mice (A53T average distance = 4031 cm, B6C3 average distance = 3000 cm, *p* = 0.016 by two-tailed unpaired *t*-test). While the open field test did not reveal any significant difference in vertical activity (rearing) between A53T and B6C3 mice (A53T mean = 19 rears/trial, B6C3 mean = 15 rears/trial; *p* = 0.356), we did observe that A53T mice visited the center of the testing area significantly more quickly than B6C3 mice (Figure 5D), suggesting that A53T mice may be less anxious relative to similarly aged controls. These results are highly consistent with previous studies in homozygous A53T animals, which also found a hyperactive and anxiolytic phenotype in A53Ts relative to controls [61,62,63]. The A53T animals used for this study also consistently demonstrated reduced nesting behavior, as measured by both the nesting task (Figure 5E) and routine observations of A53T and B6C3 cages. Nesting is an intrinsic behavior in both male and female mice [64], and throughout this study B6C3 animals of both sexes universally made deep, well-defined nests that utilized all or most of the available nesting material. By contrast, A53T animals demonstrated little or no nest-building in both the nesting task and in their normal home cages. This behavioral change emerged at a very young age in A53T mice (~2–3 mo), and aligns with previous nesting results in the A53T model [61]. Finally, we observed significantly more limb clasping in A53T mice compared with B6C3 mice (Figure 5F) regardless of the age of the animals. In sum, our results indicate that A53T mice develop a variety of subtle but consistent behavioral abnormalities early in life, which then persist without worsening until the emergence of the fatal late-stage ataxia that is characteristic of this model.

### 2.5. A53T Mice Show Increased [^18^F]ROStrace Signal and Mortality following Lipopolysaccharide Injection

**Figure 6:**
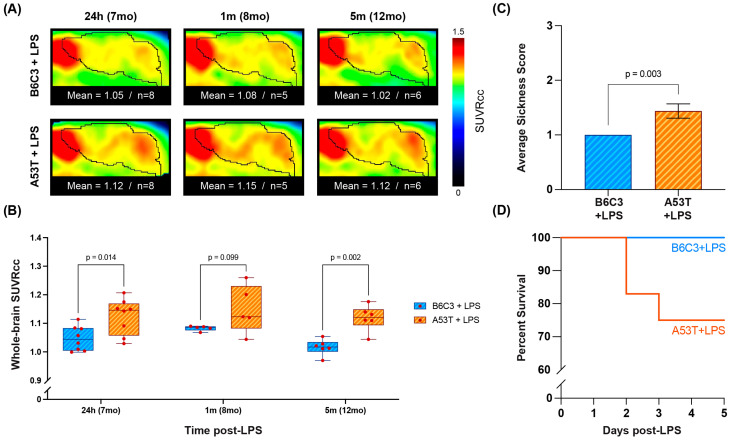
A53T animals show increased [^18^F]ROStrace signal and mortality after a single injection of LPS. (**A**) Average brain [^18^F]ROStrace images from LPS-injected B6C3 (**top**) and A53T (**bottom**) mice 24 h, 1 m, or 5 m after injection (performed at 7 mo). Colors represent CC-normalized PET signal (SUVRcc), and mean SUVRcc values and corresponding sample sizes are shown below each image. (**B**) Whole-brain SUVRcc values in LPS-injected B6C3 and A53T mice at each timepoint. *p*-values were calculated using multiple 2-tailed Welch *t*-tests with the two-stage step-up method of Benjamini, Krieger, and Yekutieli for multiple comparison correction. (**C**) Average sickness scores in LPS-injected B6C3 and A53T mice 24 h post-injection. LPS-injected A53T animals showed significantly higher sickness scores than LPS-injected B6C3 animals as calculated by two-tailed Welch’s *t*-test. (**D**) Percent survival for LPS-injected B6C3 and A53T animals 1–5 days post-injection.

It has previously been demonstrated that a single intraperitoneal (I.P.) injection of lipopolysaccharide (LPS) initiates ROS-dependent neuroinflammation and nigrostriatal degeneration in 7 mo A53T mice [38]. As such, we next sought to determine whether [^18^F]ROStrace PET imaging is sensitive enough to detect the increased susceptibility of A53T mice to a PD-related environmental challenge. To this end, we injected 7 mo A53T and B6C3 mice with LPS (5 mg/kg I.P.) and then imaged the animals with [^18^F]ROStrace either 24 h, 1 m, or 5 m after injection (Figure 6). As in the uninjected animals, LPS-injected A53T mice showed higher whole-brain average SUVRcc than LPS-injected B6C3 mice (Figure 6A). Moreover, the mean SUVRcc in LPS-injected A53Ts stayed relatively consistent up to 5 m post-injection, whereas the mean SUVRcc in LPS-injected B6C3s decreased over time (Figure 6B). Interestingly, while previous studies have reported that acute LPS-induced inflammation is similar in A53T and B6C3 animals [38], we found significant A53T-specific elevations in [^18^F]ROStrace signal as early as 24 h post-injection, suggesting that increased ROS production is an early consequence of LPS challenge in 7 mo A53T brain. During this acute phase (up to 5 days post-injection), LPS-injected A53T animals also showed higher average sickness scores and a 25% increase in mortality compared with LPS-injected B6C3 animals (Figure 6C,D). Taken together, these results suggest that early and persistent increases in [^18^F]ROStrace signal are associated with increased vulnerability of A53T animals to inflammatory insult.

## 3. Discussion

Oxidative stress is thought to be both a cause and a consequence of aSyn aggregation in the synucleinopathies. Yet despite the putative importance of oxidative stress in the pathogenesis of PD, MSA, and other prevalent neurodegenerative disorders, noninvasive methods for quantifying ROS production in these disorders have proven elusive. In this study, we used PET imaging with the ROS-sensitive radiotracer [^18^F]ROStrace to detect increased oxidative stress in the brains of living A53T mice, which demonstrate aSyn aggregation similar to that seen in human synucleinopathy (Figure 1). A53T-specific increases in [^18^F]ROStrace signal emerged relatively early in disease progression (6–8 mo) and became more widespread within the brain as the animals aged (Figure 2). These in vivo PET results were supported by 3NT staining experiments, which revealed significant and progressive increases in tyrosine nitration in A53T animals relative to age-matched wild-type controls (Figure 3). Similar trends were observed in the ps129 staining results, which showed a progressive transition from sparse, diffuse, and primarily perikaryal aggregates in 6–8 mo A53T animals to bright, distinct, and neurite-like pathology that affected most major brain regions in 12 mo A53T animals (Figure 4). Moreover, A53T-specific increases in [^18^F]ROStrace signal—and accompanying increases in 3NT and ps129 staining—were detectable during the subacute phase of disease progression (Figure 5), suggesting that small but significant elevations in ROS production are a relatively early and long-lasting feature of this model. Finally, A53T mice showed increased [^18^F]ROStrace signal and decreased survival following a single I.P. injection of LPS (Figure 6), suggesting that [^18^F]ROStrace can detect the increased vulnerability of A53T animals to LPS-induced neurodegeneration [38]. Together, these results support the idea that aSyn aggregation is associated with elevated ROS production during synucleinopathy and suggest that [^18^F]ROStrace PET imaging holds promise as a means of detecting aSyn-associated oxidative stress noninvasively.

While our PET, 3NT, and ps129 staining results strongly suggest that aSyn aggregation is associated with elevated ROS production in A53T mice, the vast majority of our A53T animals did not show evidence of significant neuroinflammation or behavioral decline, regardless of their age. Indeed, robust microglial activation and late-stage motor symptom development (i.e., weight loss, paralysis, etc.) were only observed in a handful of 12–16 mo A53T mice, with the remainder showing minimal glial activation or behavioral decline no matter the timepoint (Figure S4). These observations point towards the existence of at least two distinct phases of disease progression in A53T mice. In the first phase, sparse and diffuse aSyn pathology is accompanied by minimal microglial activation and modest but significant behavioral disruptions that do not worsen over time. During this subacute phase, our [^18^F]ROStrace and 3NT results indicate that ROS production is nonetheless elevated in A53T animals. By contrast, the later-stage, ‘acute’ phase of disease progression is characterized by the accumulation of Triton-insoluble, neuritic aSyn pathology that affects most major brain regions, alongside robust microglial activation and the rapid development of progressively severe and ultimately fatal gross motor symptoms. The sudden and catastrophic onset of this late-stage disease has been noted throughout the existence of the A53T model [36], yet it remains unclear what factor or factors are ultimately responsible for the rapid shift from sub-acute to acute disease. In light of this uncertainty, our [^18^F]ROStrace PET results are particularly interesting, as they show that significant and progressive increases in aSyn-associated oxidative stress precede the onset of acute disease by a substantial margin. Since oxidizing conditions are known to promote aSyn aggregation [26] and cell-to-cell transmission [24], such long-term increases in ROS production may accelerate the formation of insoluble aSyn lesions in particularly vulnerable populations of cells. Alternatively, chronic increases in oxidative stress could predispose A53T animals towards developing the disease later in life by interfering with intracellular antioxidant defense systems and reducing the animal’s ability to respond to future inflammatory insults. This putative mechanism is supported by our LPS results, which suggest that middle-aged A53T mice are both more susceptible to LPS-induced neuroinflammation and less able to recover from that inflammation over time.

In human PD, it is increasingly recognized that the presymptomatic (i.e., prodromal) phase of disease is likely to be a key window for outcome-altering interventions, as cardinal motor symptoms typically do not develop until substantial neurodegeneration has already occurred [20]. The current PET, staining, and behavioral results indicate that A53T mice also experience a prolonged ‘prodromal-like’ disease phase, characterized by subtle but consistent behavioral alterations (primarily hyperactivity, limb-clasping, and reduced nesting behavior [61,62,63]) and increased oxidative stress in the absence of widespread neuroinflammation. Given that CC-normalized [^18^F]ROStrace PET imaging consistently reveals increased ROS production in A53T brain during this sub-acute phase, future studies should now seek to establish whether early interventions with antioxidants and/or drugs that target aSyn aggregation are capable of reducing aSyn-associated increases in [^18^F]ROStrace signal. To this end, several antioxidant and antibiotic drugs have already shown neuroprotective effects in rodent models of neurodegeneration, including resveratrol [62], MitoQ [56,65,66], 7-nitroindazole [67], minocycline [68], and diphenyleneiodonium [69], among others. Direct and indirect inhibitors of aSyn aggregation, such as KYP-2047 [70,71], MCC950 [72], and MT101-5 [73], have also shown beneficial effects in rodent models of synucleinopathy, as have poly (ADP-ribose) polymerase-1 (PARP-1) inhibitors like ABT-888 [74] and Veliparib [75]. While the mechanisms of action, degrees of efficacy, and side effects vary dramatically amongst these drugs (and other related drugs not listed here), all of these compounds represent promising candidates for further investigation via [^18^F]ROStrace PET. Indeed, a unique advantage of [^18^F]ROStrace PET imaging is that it enables the in vivo assessment of antioxidant and anti-inflammatory drug efficacy, such that multiple candidate therapeutics could be tested and quantitatively compared in parallel in living animals. Further, interventions with different categories of pharmaceuticals (e.g., aSyn aggregation inhibitors vs. mitochondria- or microglia-targeted therapeutics) could provide valuable insights into the primary drivers of the [^18^F]ROStrace signal during each stage of disease progression. For instance, reduced [^18^F]ROStrace retention following treatment with inhibitors of aSyn aggregation (such as KYP-2047, MCC950, or MT101-5) would further solidify the putative causative relationship between aSyn aggregation and oxidative stress. Along similar lines, future studies should also seek to include cohorts of animals that are imaged longitudinally, as such studies would enable us to more thoroughly characterize both the predictive power of [^18^F]ROStrace PET and the extent to which the [^18^F]ROStrace signal progresses over the course of synucleinopathy.

In sum, the current results indicate that PET imaging with [^18^F]ROStrace is capable of detecting aSyn-associated oxidative stress in living animals, and suggest that such oxidative stress is an early and persistent feature of synucleinopathy in the A53T mouse model. To our knowledge, this study represents the first use of PET imaging as a means of detecting oxidative stress in the context of aSyn aggregation, and as such these experiments establish [^18^F]ROStrace PET as a novel and potentially valuable tool for the noninvasive characterization of oxidative stress in preclinical models of synucleinopathy.

## 4. Materials and Methods

### 4.1. Animals

Breeding pairs of *SNCA*^A53T^ (‘A53T’) and B6C3F1/J (‘B6C3’) mice were purchased from the Jackson Laboratory (Bar Harbor, ME, USA; stock numbers 004479 and 100010, respectively) and used to establish stable colonies. All housing, breeding, and experimental procedures were performed in accordance with the NIH Guide for the Care and Use of Experimental Animals and were approved by the Animal Care and Use Committees (IACUCs) at the Children’s Hospital of Philadelphia (CHOP) and the University of Pennsylvania (when applicable).

### 4.2. Immunohistochemistry

Upon reaching their designated endpoints (shown in Figure S5), animals were anesthetized and subsequently perfused with ice-cold heparinized saline. Following perfusion, the brain was extracted, and the brain hemispheres were either snap-frozen in liquid nitrogen (left hemisphere) or drop-fixed in 4% paraformaldehyde for a minimum of 48 h (right hemisphere). Drop-fixed hemispheres were then transferred to 30% sucrose for cryoprotection and subsequently embedded in Tissue-Tek optimal cutting temperature compound (Sakura Finetek Japan Co., Ltd., Tokyo, Japan) and frozen in a slurry of dry ice and isopentane. Then, 12 μm thick sections were cut from each brain and mounted on slides for immunohistochemical staining. Slides were first rinsed with phosphate-buffered saline (PBS) and permeabilized with a 0.5% solution of Triton X-100 in PBS. Blocking was achieved via a 10 min incubation with 100 mM Glycine followed by a 1 h incubation with 10% bovine serum albumin (BSA; for rabbit primary antibodies) or Mouse on Mouse IgG Blocking Reagent (for mouse primary antibodies; Vector Laboratories, Newark, CA, USA, stock no. MKB-2213-1). Blocking reagents were rinsed off with 1% BSA, and slides were then incubated with primary antibodies (Table S2) overnight at 4 °C. The following day, slides were rinsed with 1% BSA and then incubated with corresponding secondary antibodies for 1 h at room temperature. Following this incubation step, a subset of slides was also treated with an additional primary/secondary antibody combination (from a different host species); in these cases, both the primary and secondary antibodies were incubated for 1 h at room temperature with 1% BSA washes in between. Finally, all slides were counterstained with Hoechst (1:2000 in PBS, Thermo Fisher Scientific, Waltham, MA, USA, stock no. H3570) and mounted with VectaShield Antifade Mounting Media (Vector Laboratories, Newark, CA, USA, stock no. 101098-042).

Completed slides were imaged using a Zeiss Axio Observer Microscope (Carl Zeiss AG, Oberkochen, Germany), and panoramic whole-brain images were collected using a Keyence BZ-X810 Microscope (Keyence Corporation, Osaka, Japan). For quantification, 3–5 20× images were acquired per region per animal using the Zeiss microscope. In all quantification images, red, green, and blue channels were acquired, and antibody-specific Fiji scripts were used to quantify the positive staining in each image (Fiji version 2.14.0/1.54f). Fiji scripts are available at the following GitHub repository: https://github.com/Evangall/fiji-scripts (uploaded/accessed on 27 November 2023). In all cases where staining was quantified, staining and acquisition parameters were kept identical across groups being compared (e.g., 3NT staining was performed the same way in all animals, and 3NT images were acquired and quantified the same way in all animals).

### 4.3. Dihydroethidium (DHE) Injections

Animals were weighed prior to DHE preparation, and the appropriate amount of DHE solid (20 mg/kg) was then dissolved in DMSO (10 µL DMSO/mg DHE) under low-light conditions. The DHE/DMSO solution was then diluted to 1:100 with a 50/50 mixture of ethanol and Tween 20, and the resulting solution was further diluted to 1:1000 with sterile saline. The final DHE solution (concentration = 1 mg/mL) was then injected I.P. under low-light conditions. For DHE imaging and quantification, animals were sacrificed, perfused with PBS and 4% paraformaldehyde, and dissected 2 h post-injection. Brain hemispheres were then drop-fixed in 4% paraformaldehyde for 2 h and subsequently submerged in 10% sucrose overnight. The brains were then embedded and sectioned as previously described. Following sectioning, slides were warmed to room temperature, rinsed with PBS, stained with Hoechst 1:2000 for 1 min, and immediately mounted and imaged using the Zeiss microscope (as DHE was not used to quantify superoxide in this study, confocal imaging with specific excitation and emission wavelengths was not necessary [76]). To maximize the DHE signal and prevent photobleaching, all preparations of DHE and DHE-injected tissue (e.g., dissection, perfusion, embedding, sectioning, etc.) were performed under low-light conditions.

### 4.4. Pseudo-Reference Region Identification

To identify the brain region least affected by aSyn-associated oxidative stress in presymptomatic A53T mice without relying on PET imaging, immunohistochemistry was performed on sagittal and coronal brain sections from 8, 12, and 16 mo A53T and B6C3 animals (Figure S1). ROS levels in both genotypes were first visualized via DHE. Whole-brain fluorescent imaging of DHE-injected brain sections revealed a notable lack of DHE fluorescence in corpus callosum (CC), indicating that ROS production in CC is relatively low compared with other major brain structures (Figure S1A,B). To probe whether the low DHE signal in CC was associated with aSyn aggregation, additional brain sections were immunostained with ps129. While ps129-positive aSyn inclusions were widespread in A53T brain sections at all timepoints, CC consistently showed minimal ps129 fluorescence (Figure S1C,D), suggesting that CC remains largely unaffected by phosphorylated aSyn pathology throughout the majority of the A53T lifespan. Tissue sections from presymptomatic A53T animals were next stained with the antibody A11 (Figure S1E,F), which was developed against β-amyloid (Aβ) oligomers but which also detects soluble aSyn oligomers [77,78]. This staining again revealed low A11 signal in CC, indicating that CC contains relatively low concentrations of aSyn oligomers. Moreover, in sections that were co-stained with DHE and either ps129 or A11, DHE did not colocalize with either antibody in CC (Figure S1G,H), further supporting the idea that the low levels of DHE fluorescence observed in CC are not associated with the accumulation of oligomeric or phosphorylated aSyn. By contrast, A53T and B6C3 mice both showed robust ionized calcium-binding adaptor molecule 1 (Iba1) staining in CC (Figure S1I), suggesting that the DHE fluorescence observed in CC could be attributable to microglial ROS.

Since the IHC results collectively indicated that CC is spared from aSyn pathology and aSyn-associated oxidative stress in presymptomatic A53T animals, we next asked whether CC also shows low [^18^F]ROStrace signal in presymptomatic A53Ts. Towards this end, we first performed ex vivo autoradiography on A53T and B6C3 brain sections immediately following PET imaging with [^18^F]ROStrace. As expected, CC showed minimal [^18^F]ROStrace retention in both A53T and B6C3 brain tissue (representative image shown in Figure S1J). Likewise, the average [^18^F]ROStrace PET signal in CC was the lowest of any brain region examined (Figure S1K,L) as measured in standardized uptake value (SUV), which normalizes the [^18^F]ROStrace signal to account for differences in body weight and injected dose. The average SUV in CC also did not differ significantly across genotypes or timepoints (*p* = 0.286 by one-way ANOVA), indicating that signal variability in this region is relatively low compared with other major brain structures. Taken together, these results demonstrate that the corpus callosum is the most appropriate pseudo-reference region for quantification of [^18^F]ROStrace signal in A53T mice.

### 4.5. Preparation of [^18^F]ROStrace

Prior to each set of PET scans, [^18^F]ROStrace was prepared as previously described [34]. Synthesis was performed using a Trasis AllInOne radiochemistry synthesizer (Liège, Belgium), and the final product was diluted with 0.6 mL of ethanol, 0.1% ascorbic acid, and 6 mL of sterile saline before being filtered through a 0.2 μM nylon filter. The radiochemical yield was 4–20%, and the specific activity was 74 GBq/mol.

### 4.6. PET Imaging

A simplified diagram showing the time course of PET imaging experiments is shown in Figure S5. In all experiments, imaging was performed at one of two different sites. At CHOP, imaging was performed using a Siemens Inveon PET/CT scanner (Siemens Healthineers, Erlangen, Germany), whereas at Penn, imaging was performed using a Molecubes β-Cube PET scanner and X-Cube CT scanner (Molecubes, Ghent, Belgium). At both sites, animals were weighed prior to each scan and subsequently anesthetized using 1.5–2.5% isoflurane. A needle with an attached catheter was then inserted into the lateral tail vein and taped in place, after which the animals were transferred to heated beds in the PET scanners and injected with 200–300μCi of [^18^F]ROStrace. Dynamic PET images were acquired for 60 min following injection, with 5 min (Molecubes) or 10 min (Inveon) CT acquisitions following each PET scan. Anesthesia was maintained throughout both scans via a nose cone delivering 0.7–1.4% isoflurane, and each animal’s breath rate and temperature were also monitored continuously to maintain a stable scanning environment. Residual activity in the syringe was measured immediately after the injection, and residual activity in the needle and catheter was measured at the conclusion of each scan. These measurements were decay-corrected and used to calculate the final injected dose for each animal. Following each set of scans, all PET and CT images were reconstructed using manufacturer-supplied reconstruction software.

### 4.7. PET Image Analysis

All PET and CT image analysis was performed using the Pmod software package (version 3.7, PMOD Technologies Ltd., Zurich, Switzerland). Reconstructed dynamic PET images were first coregistered to their corresponding CT images using Pmod’s automated rigid-body alignment tool with 6 degrees of freedom. Once the coregistration was checked and adjusted if necessary, each CT image was manually scaled, rotated, and translated to align with the M. Mirrione T2-weighted mouse brain MRI and accompanying atlas [47]. The resulting transformation was then applied to the corresponding PET image to align it with both the CT image and the brain atlas. In its unmodified state, the Mirrione mouse brain atlas includes volumes of interest (VOIs) for 14 brain regions: striatum, cortex, hippocampus, thalamus, cerebellum, basal forebrain and septum, hypothalamus, amygdala, brainstem, central grey, superior colliculi, olfactory bulbs, midbrain, and inferior colliculi. For this project, a corpus callosum (CC) VOI was also added to the Mirrione atlas by manually tracing the CC on each coronal slice of the Mirrione T2 mouse brain MRI. The resulting modified atlas was used to calculate whole-brain and subregion-specific PET results for the remainder of the project. Notably, the CC VOI was created completely independently from the PET and CT datasets, as the VOI was drawn on an MR image that comes included with Pmod. As such, the creation of the CC VOI was not influenced in any way by the PET or CT images.

Voxel-wise statistical comparisons were performed on SUVRcc images using Statistical Parametric Mapping version 12 (Wellcome Centre for Human Neuroimaging, UCL Queen Square Institute of Neurology, London, UK) with the SPMMouse toolbox [79] implemented in MATLAB R2017b (MathWorks Inc., Natick, MA, USA).

### 4.8. LPS Injection and Sickness Scoring

LPS injections and assessments of LPS-induced neurotoxicity were performed as previously described [34]. Briefly, 7 mo A53T and B6C3 animals were injected intraperitoneally with 5 mg of LPS/kg (MilliporeSigma, Burlington, MA, USA, product number L2630). Sickness scoring was performed 24 h post-injection and entailed visual identification of (1) hunched posture, (2) neglect of grooming, (3) lack of movement, (4) piloerection, and (5) discharge from the eyes. Each symptom was categorized as either absent (0), mild (1), or severe (2), and the final sickness score was calculated based on the sum of the individual symptom scores. Summed symptom scores of 0–2 were assigned a 0, scores of 3 or 4 were assigned a 1, scores of 5–6 were assigned a 2, scores of 7–8 were assigned a 3, and scores of 9–10 were assigned a 4. In this study, no animal was assigned a score of 0 or 4; the maximum score assigned was 3 (given to two A53Ts). Sample sizes for sickness scoring were as follows: A53T *n* = 24, B6C3 *n* = 17.

### 4.9. Behavioral Assessments

#### 4.9.1. Clasping

Clasping was evaluated during routine cage checks every 2–3 days. Animals were lifted by their tail and suspended ~12 inches from the cage floor for ~10 s. The animals were then slowly lowered back towards the cage floor, and limb clasping behavior was quantified based on the following scheme: 0 = no clasping; 1 = consistent clasping of one limb, or intermittent clasping of two limbs; 2 = consistent clasping of two limbs; 3 = consistent clasping of all limbs. The final outcome measure for each animal was the average clasping score across the animal’s entire lifespan (average of ~75 observations per animal).

#### 4.9.2. Open Field Task

For the open field task, mice were placed in an open field arena (42 cm × 42 cm) and allowed to behave freely for 15 min. Each trial was recorded via an overhead camera, and EthoVision XT software (Version 15.0, Noldus Information Technology, Wageningen, The Netherlands) was used to quantify a variety of behavioral outcome measures, including mean velocity, total distance traveled, percent of the trial spent not moving, percent of the trial spent in the center zone, latency to the center zone, average distance from the center zone, and percent of the trial spent in the contracted body state. Animals were allowed to acclimate to the testing room for at least one hour prior to testing.

#### 4.9.3. Nesting Task

For the nesting task, mice were individually placed in new, clean cages containing one square of intact nesting material (i.e., ‘nestlet’; approx. 3 g each) one hour before the beginning of the 12 h dark cycle. The mice were housed in these cages for 14 h with food and water provided ad libitum, after which they were returned to their home cages. At the conclusion of each trial, nesting behavior was assessed both quantitatively and qualitatively. For nestlet quantification, intact nestlets were weighed before and after the trial, and these measurements were used to calculate the percent of the original nestlet used by the mouse to build a nest. For qualitative assessment of nesting, a condition-blinded observer evaluated each nest and scored it on a scale from 1 to 5, with 1 representing a complete or nearly complete lack of nesting behavior (i.e., more than 90% of the nestlet left intact) and 5 representing a fully formed nest (i.e., a deep, well-defined nest with more than 90% of the original nestlet used).

#### 4.9.4. Multi-Day Behavioral and Metabolic Profiling

In a subset of animals, behavioral and metabolic activity was assessed using the Comprehensive Laboratory Animal Monitoring System (CLAMS; Columbus Instruments, Columbus, OH, USA). Mice were individually housed and allowed to acclimate for 72 h prior to testing, after which they were transferred to the CLAMS apparatus for 3–4 day/night cycles. During the testing sessions, the CLAMS measured respiration frequency, O_2_ consumption, CO_2_ production, respiratory exchange ratio, and heat production every 15 min. Locomotor activity was also detected by passive infrared sensors and running wheels. Throughout their time in the CLAMS, animals were checked daily, and food and water were provided ad libitum.

## Data Availability

The raw data supporting the conclusions of this article will be made available by the authors upon request.

## Supplementary Materials

The following supporting information can be downloaded at: https://www.mdpi.com/article/10.3390/ijms25094943/s1.
