# Supplementary material for: Positron Emission Tomography with [18F]ROStrace Reveals Progressive Elevations in Oxidative Stress in a Mouse Model of Alpha-Synucleinopathy
Source: Int J Mol Sci. 2024 May 1;25(9):4943. doi: 10.3390/ijms25094943 (PMC11084161; doi:10.3390/ijms25094943)
Supplement: Supplementary file 1 [file ijms-25-04943-s001.zip › ijms-2968204-supplementary.pdf]

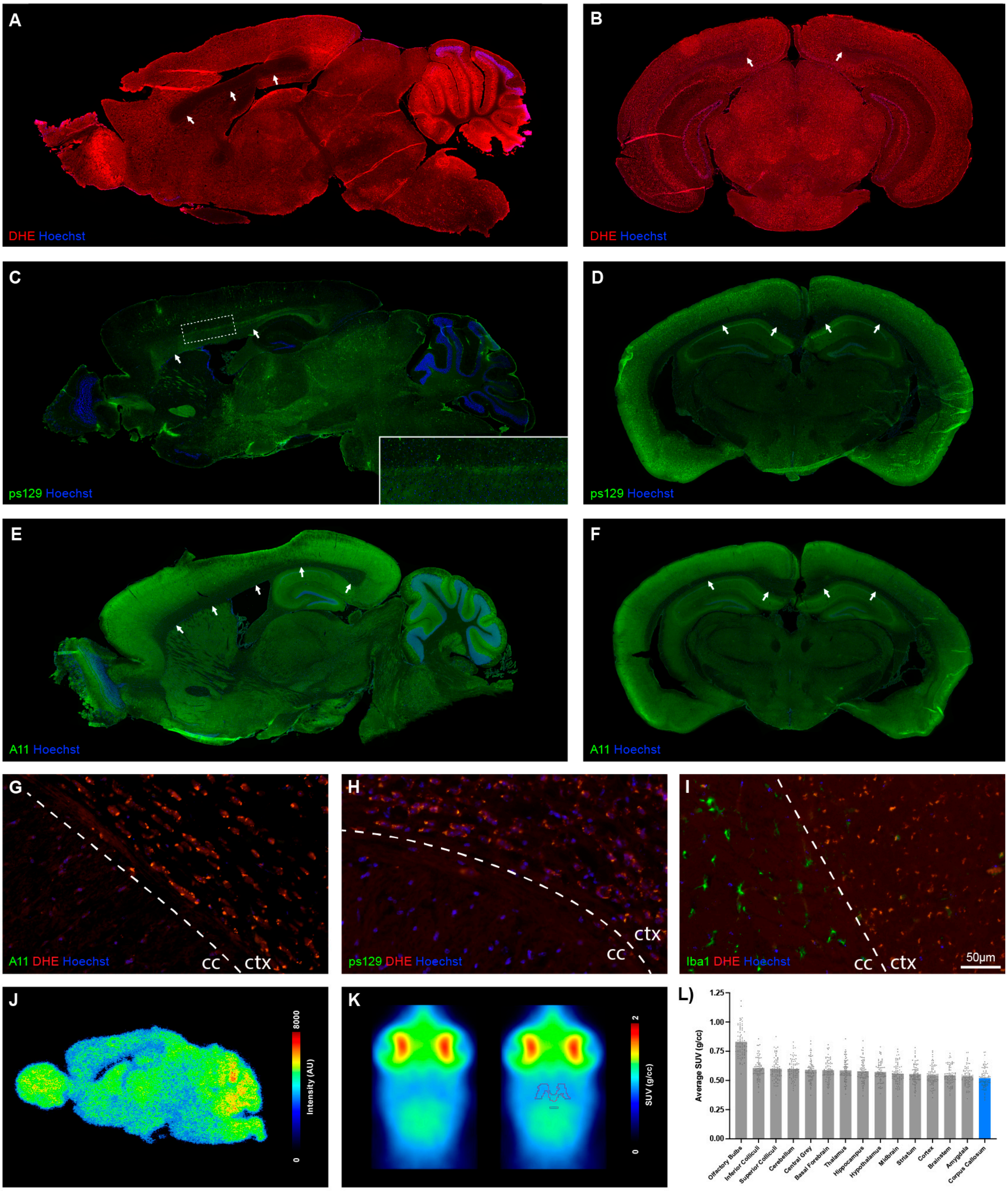

Supplementary Figure 1. Corpus callosum (CC; white arrows) is the most appropriate pseudo-reference region for quantification of brain [18F]ROStrace signal in A53T and B6C3 mice. (**A-B**) Representative fluorescent images showing the distribution of the ROS-sensitive dye dihydroethidium (DHE; 20mg/kg I.P.) in 12-16mo A53T mouse brain. (**C-D**) Sagittal and coronal A53T brain sections stained with phospho-serine 129 (ps129; 1:500), which selectively recognizes phosphorylated aSyn. The inset in panel C shows a higher-magnification image of cortex (top) and CC (bottom), and the dashed outline in the whole-brain image shows where this image was

captured. Note that the bright green puncta visible in cortex are not observed in CC. (**E-F**) Representative images of sagittal and coronal A53T brain sections stained with the soluble oligomer-specific antibody A11 (1:200). (**G-I**) Representative 20x images of 12mo A53T brain sections co-stained with A11 and DHE (G), ps129 and DHE (H), and Iba1 (microglia) and DHE (I). A11, ps129, and Iba1 all colocalized with DHE in cortex (ctx), but only Iba1 showed specific staining in corpus callosum. In all fluorescence images, cell nuclei are visualized via Hoechst (1:2000). (**J**) Representative *ex-vivo* autoradiograph from a 12mo A53T male (**K**). Representative [^{18}F]ROStrace PET images with and without the CC volume of interest (VOI). (**K**) Average standardized uptake values (SUVs) in each of the 15 VOIs in our mouse brain atlas. CC (shown in blue) had the lowest average SUV of all regions (n=78).

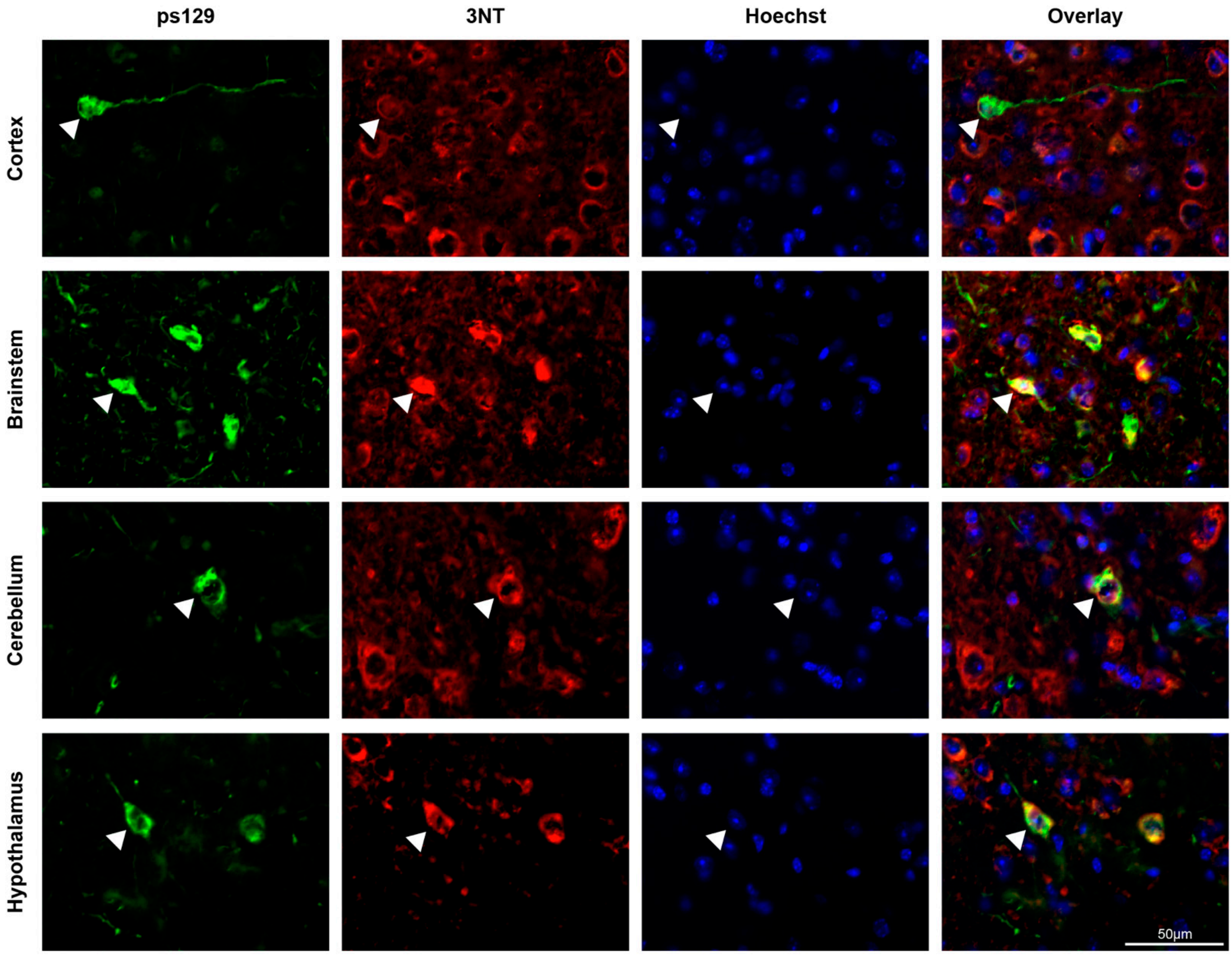

Supplementary Figure 2. Phosphorylated aSyn pathology colocalizes with 3NT-positive cells in 12mo A53T brain. The images shown here are higher magnification versions of the 12mo A53T images from Figure 4. In all images, arrows show examples of overlap between ps129 (green; phosphorylated aSyn pathology) and 3NT (red; evidence of oxidative stress). In all brain regions, ps129 pathology was consistently found in 3NT-positive cells.

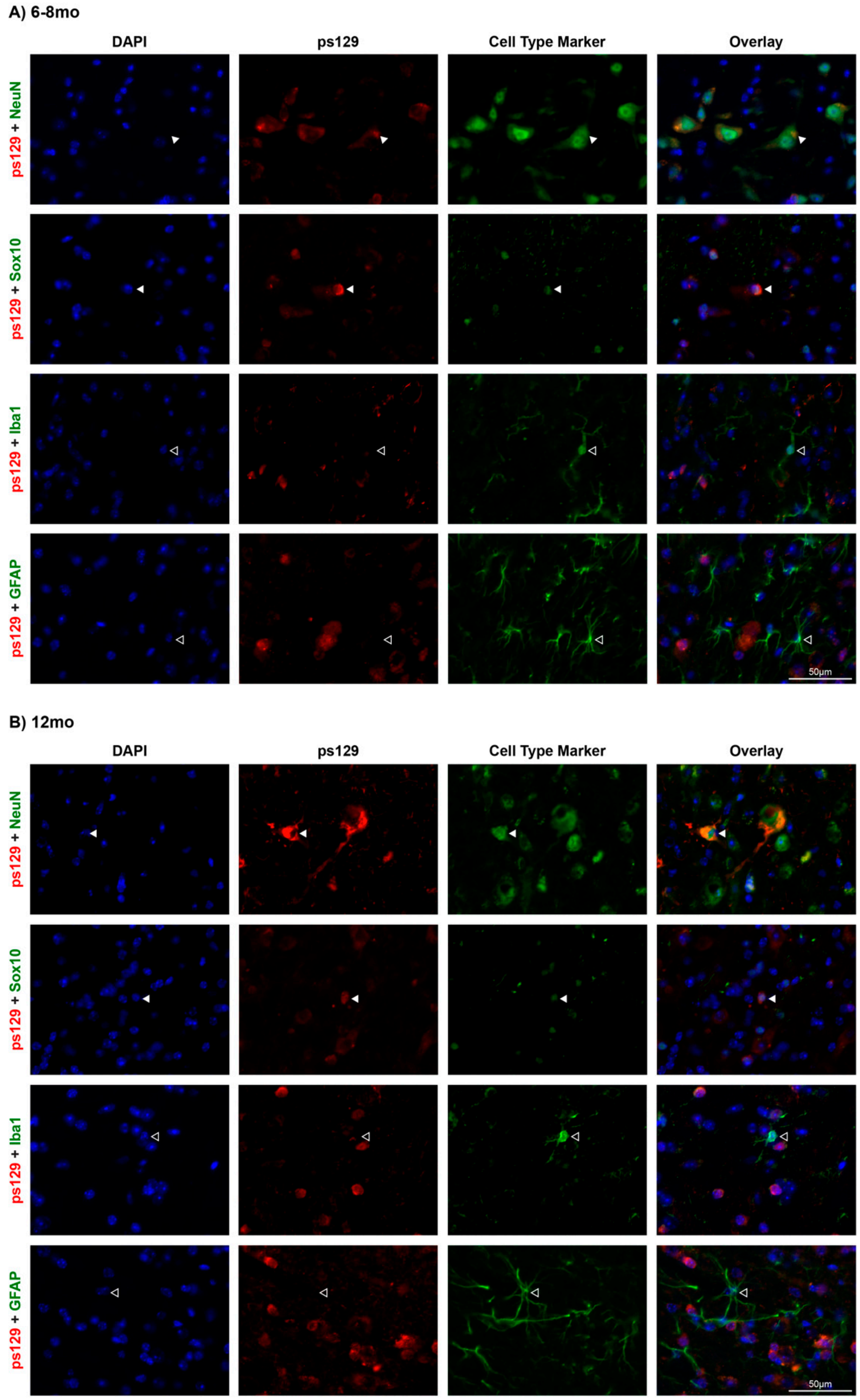

Supplementary Figure 3. Phosphorylated aSyn aggregates are primarily found in neurons. All panels show representative 20x fluorescent images from the brainstems of 6-8mo (**A**) and 12mo (**B**) A53T mice. In each row of images, phosphorylated aSyn is shown in red (ps129 1:500), nuclei are shown in blue (Hoechst 1:2000), and cell type-specific markers are shown in green (1:200 for all). Top row = ps129+NeuN (neurons); 2nd row = ps129+sox10 (oligodendrocytes); 3rd row = ps129+iba1 (microglia); bottom row = ps129+GFAP (astrocytes). Within each row, individual channels are shown in separate columns, with the last column showing an overlay of all 3 channels. Across all panels, solid arrowheads indicate examples of colocalization between the red and green channels, while outlined

arrowheads indicate cells that do not show red/green colocalization. Regardless of timepoint, most ps129 was found to be localized to neurons, with a smaller fraction being found in oligodendrocytes. Minimal ps129 signal was observed in microglia, and zero ps129 signal was observed in astrocytes.

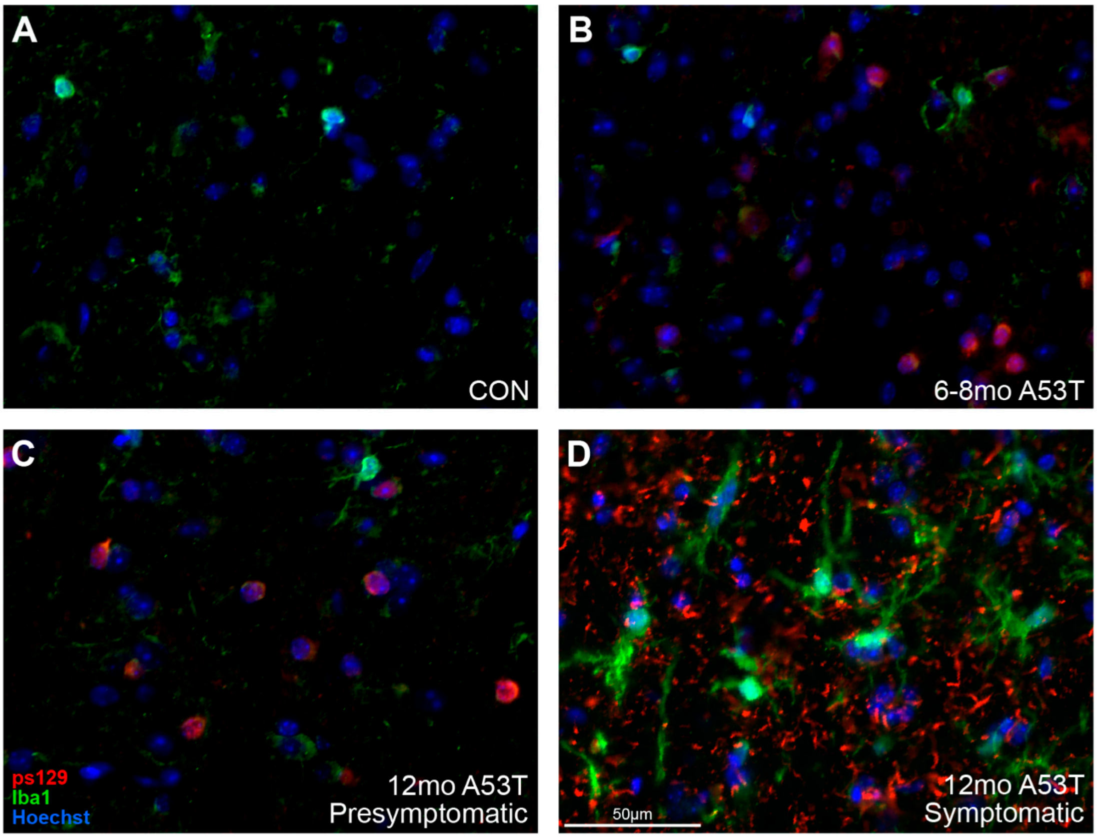

Supplementary Figure 4. Presymptomatic A53T animals show minimal microglial activation regardless of age. All panels show representative 20x fluorescent images from brainstems of 12mo B6C3 (**A**), 6-8mo A53T (**B**), 12mo presymptomatic A53T (**C**), and 12mo symptomatic A53T (**D**) mice. In each panel, red shows phosphorylated aSyn (ps129 1:500), green shows microglia (Iba1 1:200), and blue shows cell nuclei (Hoechst 1:2000). Activated microglia were only observed in symptomatic A53T mice (D), which were not the focus of this study and which were excluded from the PET dataset.

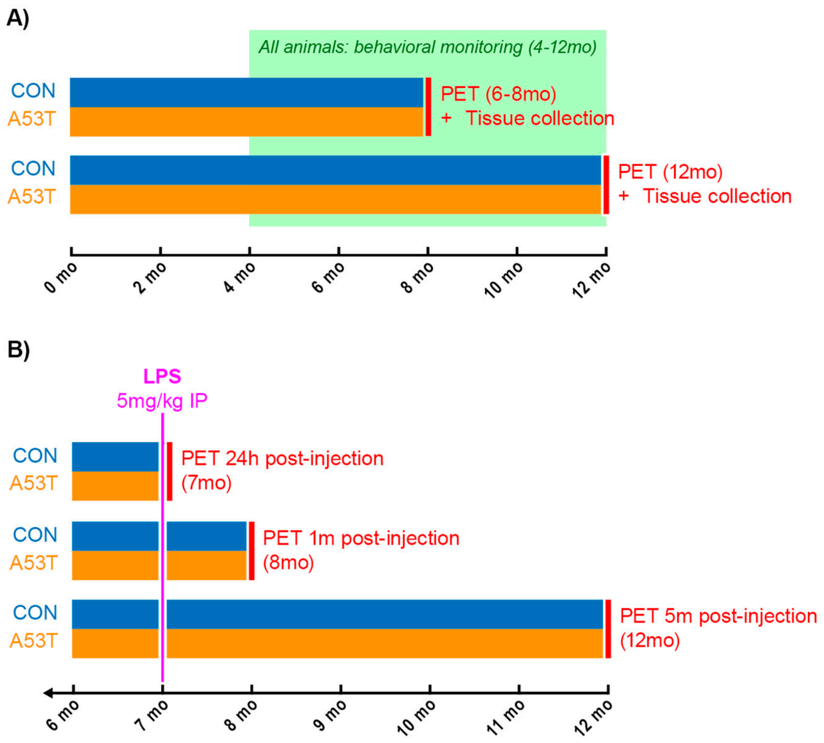

Supplementary Figure 5. Experimental design. (**A**) Simplified diagram showing the experimental workflow for non-LPS-injected animals. Cohorts of A53T and control mice of both sexes were imaged either between the ages of 6 and 8 months old (mo; top) or at 12mo (bottom). Starting at 4mo, all animals also underwent periodic behavioral assessment via the assays described in section 2.4 (green box). Following PET imaging, all animals were sacrificed for tissue collection as described in section 4.2. (**B**) Equivalent workflow diagram for LPS-injected animals. LPS injection was performed at 7mo, and cohorts of LPS-injected animals were imaged with [^{18}F]ROStrace either 24h (top), 1m (middle), or 5m (bottom) post-injection. For all PET imaging experiments, sample sizes are shown in Table S1.

Supplementary Table 1. Sample sizes. Note that the sample sizes shown here refer to [^{18}F]ROStrace imaging only; other sample sizes (e.g. for staining and behavior) are noted throughout the text.

	**Total**		Female		Male		LPS (Male only)		
	**6-8mo**	**12mo**	6-8mo	12mo	6-8mo	12mo	7mo	8mo	12mo
**A53T**	**28**	**23**	5	5	23	18	8	5	6
**B6C3**	**27**	**26**	8	10	19	16	8	5	6

Supplementary Table 2. Antibodies used.

	Name	Host	Target	Application	Company	Product Number	Primary Dilution (if applicable)	Secondary Dilution (if applicable)
Primary	ps129	Rabbit	Phosphorylated aSyn	IHC	Abcam	ab51253	1:500	1:1000
	ps129	Mouse	Phosphorylated aSyn	IHC	Abcam	ab184674	1:200	1:300
	3NT	Rabbit	Nitrated tyrosine residues	IHC	MilliporeSigma	AB5411	1:200	1:300
	Iba1	Rabbit	Microglia	IHC	Fujifilm Wako	019-19741	1:200	1:300
	GFAP	Mouse	Astrocytes	IHC	MilliporeSigma	MAB360	1:300	1:500
	Sox10	Mouse	Oligodendrocytes	IHC	Santa Cruz	sc-365692	1:200	1:300
	A11	Rabbit	Soluble protein oligomers	IHC	ThermoFisher	AHB0052	1:200	1:300
	aSyn	Rabbit	Human and mouse aSyn	WB	Proteintech	10842-1-AP	1:1000	1:20000
	NSE	Rabbit	Neuron-specific enolase	WB	Abcam	ab53025	1:4000	1:20000
	Vimentin	Rabbit	Vimentin	WB	Abcam	ab92547	1:1000	1:20000
Secondary	Alexa 488	Goat	Anti-rabbit	IHC	Abcam	ab150077		
	Alexa 488	Goat	Anti-mouse	IHC	Abcam	ab150113		
	Alexa 568	Goat	Anti-rabbit	IHC	Abcam	ab175471		
	Alexa 568	Goat	Anti-mouse	IHC	Abcam	ab175473		
	IRDye 800CW	Donkey	Anti-rabbit	WB	LI-COR	926-32213		
	IRDye 800CW	Donkey	Anti-mouse	WB	LI-COR	926-32212		
